# Knowledge, Attitudes, and Practices Regarding Monkeypox (Mpox) Among Medical Students in Telangana, India: A Cross-Sectional Analysis

**DOI:** 10.7759/cureus.109945

**Published:** 2026-05-30

**Authors:** Shreya Veggalam, Maheshwar Reddy Kandi, Vighnesh Devulapalli, Sriguna Bannur, Nethra Chittiprol, Niloufer Ahamed, Venkataramana Kandi

**Affiliations:** 1 Medicine, Prathima Institute of Medical Sciences, Karimnagar, IND; 2 Microbiology, Government Medical College, Sircilla, IND; 3 Community Medicine, North Bengal Medical College, Darjeeling, IND; 4 Health Sciences, Cypress Bay High School, Weston, USA; 5 Medicine, Mahavir Institute of Medical Sciences, Hyderabad, IND; 6 Clinical Microbiology, Prathima Institute of Medical Sciences, Karimnagar, IND

**Keywords:** bachelor of medicine and bachelor of surgery (mbbs), knowledge attitudes and practices (kap), mbbs students, mpox, mpox virus (mpxv), physicians, postgraduates

## Abstract

Introduction

Monkeypox (Mpox) is a reemerging zoonotic disease caused by the monkeypox virus (MPXV). Mpox was declared a Public Health Emergency of International Concern by the WHO. Medical students, as future healthcare providers, are critical to outbreak preparedness. This study assessed knowledge, attitudes, and practices (KAP) concerning mpox among medical students and professionals in Telangana, India.

Methods

A cross-sectional descriptive study was conducted at Prathima Institute of Medical Sciences between December 2024 and May 2025. A total of 219 participants, including 205 Bachelor of Medicine and Bachelor of Surgery (MBBS) students, six postgraduates (PGs), five interns, and three physicians, completed a validated 32-item questionnaire comprising 16 knowledge, eight attitude, and eight practice items. The validity and reliability of the KAP questionnaire were confirmed (Cronbach’s α: 0.76). Statistical analyses included the Kruskal-Wallis H test, Dunn’s post hoc test with Bonferroni correction, the Mann-Whitney U test, and Spearman’s correlation, with η² effect sizes and 95% CIs reported.

Results

Participants were predominantly female (65.3%) and aged 20-22 years (52.5%). Mean scores were as follows: knowledge, 7.26±2.65; attitude, 4.19±1.51; practice, 3.17±1.64; and total KAP, 14.62±4.49. Sex-based differences were non-significant (p>0.05). Age influenced outcomes: participants aged ≥23 years had higher attitude scores (median 5; H=7.02; p=0.030; η²=0.08; 95% CI: 0.02-0.14; Bonferroni-adjusted p=0.048 vs. 17-19 years) and practice scores (median 4; H=7.64; p=0.022; η²=0.07; 95% CI: 0.01-0.13; adjusted p=0.044). Knowledge differences across age groups were modest (H=6.94; p=0.031; η²=0.06; 95% CI: 0.01-0.12) but were not significant after correction. Educational level affected attitudes (H=6.11; p=0.048; η²=0.07; 95% CI: 0.01-0.13), with PGs and physicians scoring higher than MBBS students; practices showed a non-significant trend (p=0.097). First-year students had the lowest total KAP score (18.08±5.15), while PGs achieved the highest score (23.83±5.91). Knowledge correlated moderately with attitude (ρ=0.40, p<0.001) and weakly with practice (ρ=0.28, p<0.001); attitude correlated moderately with practice (ρ=0.33, p<0.001). Preventive practices remained consistently low across all groups.

Conclusion

Medical students in Telangana demonstrated moderate knowledge and favorable attitudes toward mpox but critically low preventive practices. Advancing age and higher educational levels were associated with improved attitudes and practices, although knowledge gains plateaued. The gap between awareness and application underscores the need for competency-based curricular reforms emphasizing hands-on infection-control training to strengthen outbreak preparedness.

## Introduction

Mpox, formerly known as monkeypox, is a zoonotic disease that has historically been endemic in parts of Africa but has since spread to other continents. The mpox virus (MPXV) belongs to the Orthopoxvirus genus within the Poxviridae family. In August 2024, the WHO identified the upsurge of mpox, a disease caused by MPXV, as a public health emergency of international concern (PHEIC) [1]. MPXV is a linear, double-stranded DNA virus. MPXV was first isolated from Macaca monkeys and was described as a “pox-like disease” by von Magnus P et al. in 1958 in Copenhagen, Denmark [2]. In the Democratic Republic of the Congo (DRC), close to Basankusu, a nine-month-old boy was the first known human case of mpox in 1970 [3]. Despite being discovered in humans more than 50 years ago, mpox’s rapid spread and evolving transmission patterns have caused major public health concerns in the wake of its recent global outbreak [4]. Mpox is mostly transmitted through intimate, prolonged contact with an infected person, contaminated objects, or direct or indirect contact with an infected animal. Fever, headache, lymphadenopathy, myalgia, and a distinctive skin rash are often the first signs of mpox. Although these signs and symptoms may be similar to those of smallpox, mpox typically manifests with less severe symptoms [5].

There are two lineages of MPXV: Clade I and Clade II. Clades Ia and Ib are the two subtypes that make up Clade I, and Clades IIa and IIb are the two subtypes that make up Clade II. Clade I is thought to be more severe than Clade II because of its higher case fatality rate and significant genetic diversity [6]. International attention has been drawn to the transborder spread of mpox into non-endemic areas, including Europe and America [7]. Although the case fatality ratio (CFR) for MPXV across Clades I and II combined is approximately 4%, the virus nevertheless poses a serious health concern [8].

From January 2025 to January 2026, a total of 54,817 confirmed mpox cases and 221 deaths were reported globally, corresponding to a CFR of 0.4%. In January 2026 alone, 1,334 new cases were documented across 50 countries, with the African Region contributing 66% (881 cases). Regional trends showed an increase in Europe (+67%, 216 cases) but declines in the Americas (-54%, 172 cases), Eastern Mediterranean (-75%, one case), Western Pacific (-46%, 49 cases), and South-East Asia (-12%, 15 cases). Country-specific highlights include Guinea (2,153 cases, six deaths), Comoros (16 cases, first reported in January 2026), La Réunion (two imported cases), the United Kingdom (23 clade Ib cases), and India (one recombinant clade Ib/IIb case) [9].

So far, more than 100 cases of mpox have been reported in India, indicating continued public health relevance. On July 15, 2022, India reported the first case of mpox in the WHO South-East Asia Region (SEAR), when a man from Kerala was diagnosed with the disease after traveling from the United Arab Emirates (UAE). Clade Ib MPXV was identified in 10 mpox cases recorded in India between September 2024 and March 2025. The probability of a large outbreak is considered low, while the overall global risk remains moderate [10, 11].

Addressing mpox is essential for public health, social, and economic reasons. Outbreaks and international transmission are largely associated with increased global mobility. Individuals with compromised immune systems, children, and pregnant women are among the most susceptible groups. Children make up 39% of India’s population, and pregnant women make up 3.3%, together representing a substantial potentially vulnerable population [12]. The main priority must be to stop the spread and contain MPXV in order to avoid overtaxing the healthcare system and disrupting the economy. Although specific treatments for mpox remain limited, some antiviral therapies and supportive management approaches are available. Consequently, it is essential to sustain preparedness for emergency responses to future public health emergencies [13]. Healthcare systems have already been strained and overburdened as a result of the global COVID-19 outbreak caused by SARS-CoV-2. Under such circumstances, a new outbreak or pandemic could overburden medical facilities, threaten nations, and worsen already strained health systems.

The proficiency of healthcare professionals in recognizing mpox signs and symptoms can be assessed through knowledge evaluation. Early diagnosis is critical for containment and patient care, while improved awareness and safety measures remain essential in India. During mpox outbreaks, understanding attitudes helps identify barriers to effective interventions and fosters proactive, responsible behavior. Practices within healthcare settings reflect the translation of knowledge and attitudes into action, demonstrating preparedness and clinical conduct. Clinicians’ responses to suspected cases provide insight into the healthcare system’s readiness for future epidemics or pandemics.

This study aimed to evaluate the knowledge, attitudes, and practices (KAP) related to mpox among undergraduate medical students pursuing the Bachelor of Medicine and Bachelor of Surgery (MBBS) degree, postgraduate (PG) trainees, and physicians in Telangana, India. In accordance with the Strengthening the Reporting of Observational Studies in Epidemiology (STROBE) guidelines, we prespecified the following hypotheses: the primary hypothesis was that advanced educational levels, including senior undergraduates (UGs) and interns, would be associated with higher knowledge scores compared to junior UGs; secondary hypotheses were that older participants would demonstrate more positive attitudes toward mpox prevention and that preventive practice scores would remain consistently low across demographic groups regardless of age, sex, or educational level. We further anticipated that variations in KAP would be driven by medical education and professional status, with interns, PGs, and physicians expected to demonstrate higher knowledge, more positive attitudes toward vaccination and public health measures, and greater adherence to preventive practices compared to junior UGs. Demographic factors such as age and sex were hypothesized to be significant predictors of KAP levels, and a strong correlation was expected between self-perceived knowledge and actual clinical preparedness, with higher knowledge translating into proactive attitudes and greater compliance with established protocols for screening and notifying health authorities about suspected cases.

## Materials and methods

A cross-sectional observational descriptive study was conducted at Prathima Institute of Medical Sciences (PIMS), a private medical college and teaching hospital in Telangana with 960 beds and an annual intake of 250 MBBS students, located in a rural setting. The campus also offers 139 postgraduate seats across various specializations. The study examined KAP concerning mpox among 219 participants, including MBBS students (n=205), PG students (n=6), interns (n=5), and physicians (n=3). Purposive sampling was employed to ensure accessibility to the target population most relevant to the study objectives. MBBS students, PG trainees, interns, and physicians were prioritized as they represent groups with direct or imminent responsibility in patient care and public health preparedness. This approach allowed the study to capture KAP among those most likely to encounter mpox cases in clinical settings, while excluding populations less likely to contribute meaningful insights into clinical preparedness. All participants included in the study agreed to complete the questionnaire. Those who decided to participate provided written informed consent prior to data collection. The study was approved by the Institutional Ethics Committee of PIMS, Karimnagar (IEC/PIMS/UG/2024/12), and was carried out between December 2024 and May 2025. The study participants remained anonymous, their privacy was maintained, and their information was kept strictly confidential. The study was reported in accordance with the Strengthening the Reporting of Observational Studies in Epidemiology (STROBE) checklist (Appendix 1).

Inclusion and exclusion criteria

The study included MBBS students in their different phases (1st, 2nd, 3rd, and 4th/final year, and interns), PG students across medical specializations, and physicians affiliated with PIMS, as these groups were most relevant to assessing knowledge, attitudes, and practices concerning mpox due to their direct or imminent involvement in patient care. Individuals not formally enrolled at PIMS or not part of the teaching hospital staff, as well as those unwilling to provide informed consent, were excluded.

Sample size estimation

The following formula was used to calculate the sample size: n = Zα²(1-p)/e²p, where p (78.1%) indicates the prevalence of acceptable KAP in relation to mpox, e (7%) indicates the relative error, and Z (1.96) was computed at a 95% CI [14]. Using the information above, a final sample size of 220 was determined; however, one participant withdrew from the study, resulting in a final sample size of 219. A total of 219 participants out of the estimated 220 completed the survey, resulting in a 99.54% response rate.

The study questionnaire

The questionnaire was designed specifically for this study, pre-tested, and validated prior to data collection. It comprised two components: sociodemographic information and KAP related to mpox. Sociodemographic variables included participants’ current level of medical education, sex, age in completed years, and native state. The KAP section contained 32 items: 16 assessing knowledge, eight assessing attitudes, and eight assessing practices. Of these, 30 items were scored dichotomously, with “yes” responses assigned a score of 1 and “no” or “don’t know” responses assigned a score of 0. Two attitude items used Likert scales: one employed a three-point scale (“not confident,” “a little confident,” and “very confident”) scored 0, 1, and 2, respectively; the other used a five-point scale, where “strongly disagree,” “disagree,” and “neutral” were scored 0, “agree” was scored 1, and “strongly agree” was scored 2. Knowledge was thus evaluated by assigning one point for each correct response, attitudes were measured using Likert-scale items, with some dichotomized by mean score as above mean = positive and below mean = negative, and practices were assessed through responses reflecting the frequency of behaviors (e.g., “always” to “never”).

Interpretation of scores

Knowledge was assessed on a maximum score of 15, with performance categorized as good/adequate (12-15; ≥75-80%), moderate/fair (8-11; 50-74%), and poor/inadequate (0-7; <50%). Attitudes were measured on a maximum score of 8 and classified as positive/acceptable (6-8; ≥70-75%) or negative/inadequate (0-4; <50-60%), with a borderline/fair range often considered at 5. Practices were similarly evaluated on a maximum score of 8, with good/proper practices defined as 6-8 (≥70-75%), fair/moderate practices as 4-5 (50-74%), and poor/inadequate practices as 0-3 (<50%).

Validation and reliability

The questionnaire was pilot tested on 10 persons. An internal panel of three specialists from Microbiology, Community Medicine, and General Medicine evaluated the tool’s clarity, relevance, and comprehensiveness. After expert assessment, Cronbach’s alpha (α) coefficient was used to assess the internal consistency and reliability of the KAP questionnaire. The computed α values were 0.82 for knowledge (good), 0.74 for attitude (acceptable), 0.68 for practice (fair), and α=0.76 for the complete questionnaire (acceptable), indicating that this exploratory study had acceptable to moderate internal consistency. Exploratory factor analysis (EFA) was used to further investigate construct validity. The data-gathering process began when potential volunteers were informed about the project. Each willing participant provided written informed consent before beginning to complete the questionnaire. Data were gathered using Google Forms (Google LLC) (see Appendices). There were no missing values for the key KAP variables among the 219 responses.

Statistical analysis

The data gathered were entered into a Microsoft Office 2019 Excel spreadsheet (Microsoft Corp., Redmond, WA, USA). Following that, IBM SPSS Statistics for Windows, Version 20 (Released 2011; IBM Corp., Armonk, NY, USA) was used to conduct statistical analysis and draw conclusions. Descriptive statistics represented demographic variables as frequencies and percentages. Continuous variables, including KAP scores, were described using means, SDs, and medians. The normality of KAP scores was evaluated using the Shapiro-Wilk test (see Appendices). Because all scores showed a non-normal distribution, non-parametric approaches were used for all subsequent inferential analyses. The Kruskal-Wallis H test was used to compare KAP scores across multiple independent groups, specifically educational levels and age groups. When a significant difference was identified, pairwise comparisons were conducted using Dunn’s post hoc test with Bonferroni correction. The Mann-Whitney U test was used to compare two independent groups, such as genders. Spearman’s rank correlation coefficient (ρ) was used to evaluate the relationships between knowledge, attitude, and practice areas, given the non-normal distribution of scores. Effect sizes were reported as η², with 95% CIs calculated to quantify the magnitude of differences. All statistical tests were two-tailed, with a probability (p) value of less than 0.05 indicating statistical significance.

## Results

A total of 219 medical students and professionals participated in the study. The majority were aged 20-22 years (n=115, 52.5%), followed by those aged 17-19 years (n=86, 39.3%), with only 18 participants (8.2%) aged ≥23 years. Females comprised nearly two-thirds of the sample (n=143, 65.3%), while males accounted for 34.7% (n=76). In terms of educational status, 1st-year MBBS students represented half of the cohort (n=110, 50.2%), followed by 2nd-year (n=45, 20.5%) and 3rd-year students (n=41, 18.7%). Smaller proportions included 4th-year students (n=9, 4.1%), PGs (n=6, 2.7%), interns (n=5, 2.3%), and physicians (n=3, 1.4%). Geographically, most respondents were residents of Telangana (n=194, 88.6%). The remainder were from Andhra Pradesh (n=9, 4.1%), Maharashtra (n=8, 3.7%), and other states, including Rajasthan, Kerala, Tamil Nadu, Bihar, Uttar Pradesh, and one other state, each representing ≤1% of the sample (Table 1).

**Table 1:** Sociodemographic characteristics of the study participants (n=219). MBBS: Bachelor of Medicine and Bachelor of Surgery.

Characteristic	Category	Frequency (n)	Percentage (%)
Age (years)	17-19	86	39.3
	20-22	115	52.5
	≥23	18	8.2
Sex	Female	143	65.3
	Male	76	34.7
Medical education/professional status	1st-year MBBS	110	50.2
	2nd-year MBBS	45	20.5
	3rd-year MBBS	41	18.7
	4th-year MBBS	9	4.1
	Postgraduate	6	2.7
	Intern	5	2.3
	Physician/practitioner	3	1.4
State of residence	Telangana	194	88.6
	Andhra Pradesh	9	4.1
	Maharashtra	8	3.7
	Rajasthan	2	0.9
	Kerala	2	0.9
	Tamil Nadu	1	0.5
	Bihar	1	0.5
	Uttar Pradesh	1	0.5
	Other	1	0.5

The assessment of KAP regarding mpox among the 219 participants revealed a mean knowledge score of 7.26±2.65 (median: 8; maximum: 15), indicating a moderate level of awareness. The attitude domain showed a mean score of 4.19±1.51 (median: 4; maximum: 8), while the practice score was the lowest among the three domains at 3.17±1.64 (median: 3; maximum: 8). Overall, the cumulative KAP score for the group was 14.62±4.49 (median: 15; maximum: 31) (Table 2). The distribution of KAP scores across domains is further illustrated in Appendix 2. A box plot showing the distribution of KAP scores is provided in Appendix 3.

**Table 2:** Summary of score distribution across KAP domains among study participants (n=219). KAP: Knowledge, attitudes, and practices.

Domain	Score, mean±SD	Median score	Maximum possible score
Knowledge	7.26±2.65	8	15
Attitude	4.19±1.51	4	8
Practice	3.17±1.64	3	8
Overall KAP score	14.62±4.49	15	31

When stratified by sex, male and female participants demonstrated comparable KAP scores regarding mpox. Median scores were identical across sexes (knowledge=8, attitude=7, practice=3, total KAP=18). Males had a slightly higher mean knowledge score (7.89 vs. 7.28), while females reported marginally higher mean attitude scores (7.10 vs. 6.83). Practice scores were low in both groups (3.06±1.32 for females vs. 3.37±2.12 for males). Overall, total KAP scores were similar (17.44±4.65 for females vs. 18.09±5.61 for males) (Table 3).

**Table 3:** Comparison of KAP scores by sex among study participants (n=219). KAP: Knowledge, attitudes, and practices.

Sex	Knowledge, mean±SD	Knowledge, median	Attitude, mean±SD	Attitude, median	Practice, mean±SD	Practice, median	Total KAP score, mean±SD	Total KAP score, median
Female	7.28±2.55	8	7.10±2.07	7	3.06±1.32	3	17.44±4.65	18
Male	7.89±3.28	8	6.83±2.06	7	3.37±2.12	3	18.09±5.61	18

The Mann-Whitney U test was conducted to assess differences in KAP scores between male and female participants. Statistical analysis revealed no significant sex-based differences across any domain. Median scores were identical for both groups (knowledge=8, attitude=7, practice=3, total KAP=18). Males had a slightly higher mean knowledge score, while females had a marginally higher mean attitude score. Practice scores were similar, as were total KAP scores. None of these differences reached statistical significance, indicating that sex did not influence mpox-related KAP outcomes in this study population (Table 4). The sex-based distribution of KAP scores is shown graphically in Appendix 4.

**Table 4:** Assessment of sex-based differences in KAP scores among study participants (n=219). KAP: Knowledge, attitudes, and practices; U statistic: Mann-Whitney U statistic; p-value: Probability value. Statistical significance was assessed using the Mann-Whitney U test; p-values reflect comparisons of KAP scores by sex. A p-value of less than 0.05 was considered statistically significant.

Variable	U statistic	p-value	Significance
Knowledge score	5901	0.2927	Not significant
Attitude score	5084	0.4266	Not significant
Practice score	5468.5	0.9376	Not significant
Total KAP score	5686	0.5723	Not significant

Analysis of KAP scores across age groups revealed clear differences. Younger respondents aged 17-19 years had the lowest median knowledge (6.0) and attitude scores (9.0), along with comparatively weaker practice scores (median 3.0). Participants aged 20-22 years demonstrated higher knowledge (median 8.0) and attitude scores (median 10.0), with modestly better practices (median 3.0). The oldest group (≥23 years) showed the strongest attitudes (median 10.0) and the highest practice scores (median 4.0), while their knowledge scores (median 7.0) were comparable to those of the 20-22-year age group. Overall, these findings suggest that increasing age, and by extension, progression in medical education, is associated with more positive attitudes and improved preventive practices, although knowledge gains plateau beyond the early twenties (Table 5). The age-based distribution of KAP scores is shown in Appendix 5.

**Table 5:** Comparison of KAP scores by age group among study participants (n=219). KAP: Knowledge, attitudes, and practices.

Age group (years)	Number per group	Knowledge, mean±SD	Knowledge, median	Attitude, mean±SD	Attitude, median	Practice, mean±SD	Practice, median
17-19	86	6.23±2.59	6	8.95±2.00	9	2.79±1.46	3
20-22	115	7.19±2.32	8	9.60±1.67	10	3.36±1.72	3
≥23	18	7.28±1.90	7	10.39±1.94	10	3.78±1.63	4

Table 6 presents the comparison of KAP scores across age groups. Median knowledge scores were 7 (IQR: 6-9) for participants aged 17-19 years, 8 (IQR: 7-10) for those aged 20-22 years, and 8 (IQR: 7-11) for those aged ≥23 years. The Kruskal-Wallis test indicated a modest difference (H=6.94, p=0.031; η²=0.06, 95% CI: 0.01-0.11), but Bonferroni-adjusted post hoc comparisons did not reach statistical significance (adjusted p=0.186), reflecting only a non-significant trend toward higher knowledge in older groups. Attitude scores showed clearer differences: medians were 3 (IQR: 2-4) for ages 17-19 years, 4 (IQR: 3-5) for ages 20-22 years, and 5 (IQR: 4-6) for ages ≥23 years. The Kruskal-Wallis test confirmed statistical significance (H=7.02, p=0.030; η²=0.08, 95% CI: 0.03-0.13), with Bonferroni-adjusted comparisons indicating that older participants aged ≥23 years had significantly more positive attitudes compared to the youngest group (adjusted p=0.048). Preventive practice scores remained consistently low across all groups, with medians of 2 (IQR: 1-3) for ages 17-19 years, 3 (IQR: 2-4) for ages 20-22 years, and 4 (IQR: 3-5) for ages ≥23 years. The Kruskal-Wallis test revealed significant variation (H=7.64, p=0.022; η²=0.07, 95% CI: 0.02-0.12), and Bonferroni-adjusted comparisons showed that older participants reported significantly higher practice scores than the youngest group (adjusted p=0.044) (Table 6).

**Table 6:** Comparison of KAP domains across age groups. KAP: Knowledge, attitudes, and practices; H statistic: Kruskal-Wallis H statistic; p-value: Probability value; η²: eta-squared; adjusted p-value: Bonferroni-corrected probability value. Group differences were assessed using the Kruskal-Wallis H test for non-parametric data. Post hoc pairwise comparisons were performed using Dunn’s test with Bonferroni correction to adjust for multiple testing. Effect sizes were reported as eta-squared (η²) with 95% confidence intervals to quantify the magnitude of differences. A p-value of less than 0.05 was considered statistically significant.

Variable	Age group (years)	Median (IQR)	H statistic	p-value	Adjusted p-value (Bonferroni)	η² (effect size)	95% CI for η²
Knowledge	17-19	7 (6-9)	6.94	0.031	>0.05	0.06	0.01-0.11
	20-22	8 (7-10)					
	≥23	8 (7-11)					
Attitudes	17-19	3 (2-4)	7.02	0.03	0.048	0.08	0.03-0.13
	20-22	4 (3-5)					
	≥23	5 (4-6)					
Practices	17-19	2 (1-3)	7.64	0.022	0.044	0.07	0.02-0.12
	20-22	3 (2-4)					
	≥23	4 (3-5)					

Table 7 presents the results of Dunn’s post hoc test with Bonferroni correction for pairwise comparisons of KAP scores across age groups. Significant differences were observed between the 17-19- and 20-22-year age groups, with younger students scoring lower in knowledge (p=0.011), attitude (p=0.037), and practice (p=0.029). Comparisons between the 17-19- and ≥23-year age groups showed significant differences in attitude (p=0.031) and practice (p=0.022), but not in knowledge (p=0.169). No significant differences were found between the 20-22- and ≥23-year age groups (knowledge: p=0.839; attitude: p=0.259; practice: p=0.258). These findings indicate that the youngest age group consistently demonstrated lower KAP scores compared to older peers, while differences between the middle and oldest age groups were not statistically significant.

**Table 7:** Pairwise comparison of KAP scores across age groups (n=219). KAP: Knowledge, attitudes, and practices; p-value: Probability value. Pairwise comparisons of KAP scores between age groups were conducted using Dunn’s post hoc test with Bonferroni correction. Reported p-values indicate the significance of differences between groups. A p-value of less than 0.05 was considered statistically significant.

Age-group comparison	Knowledge p-value	Attitude p-value	Practice p-value
17-19 vs. 20-22	0.011	0.037	0.029
17-19 vs. ≥23	0.169	0.031	0.022
20-22 vs. ≥23	0.839	0.259	0.258

Table 8 summarizes the mean KAP scores across different stages of medical training. First-year students had the lowest overall KAP scores (knowledge: 6.43±2.65; attitude: 8.63±2.20; practice: 3.03±1.90; total: 18.08±5.15). Scores improved progressively among 2nd- and 3rd-year students, with 3rd-year students achieving higher knowledge (7.41±1.99) and total KAP scores (20.17±3.43). Interns demonstrated further gains (total KAP: 21.20±1.48), while PGs recorded the highest scores across all domains (knowledge: 8.33±2.16; attitude: 10.83±2.56; practice: 4.67±2.16; total: 23.83±5.91). Physicians showed relatively lower scores compared to PGs (total: 18.67±3.21) (Table 8). The graphical representation of KAP scores by educational level is provided in Appendix 6.

**Table 8:** Comparison of KAP scores by educational level among study participants (n=219). MBBS: Bachelor of Medicine and Bachelor of Surgery; KAP: Knowledge, attitudes, and practices.

Educational level (n)	Knowledge, mean±SD	Attitude, mean±SD	Practice, mean±SD	Total KAP score, mean±SD
MBBS 1st year (110)	6.43±2.65	8.63±2.20	3.03±1.90	18.08±5.15
MBBS 2nd year (45)	6.82±2.22	9.29±1.70	3.18±1.27	19.29±3.83
MBBS 3rd year (41)	7.41±1.99	9.37±2.02	3.39±1.20	20.17±3.43
MBBS 4th year (9)	7.44±1.74	8.78±1.56	2.78±1.20	19.00±3.24
Intern (5)	8.00±1.00	9.80±1.30	3.40±0.89	21.20±1.48
Postgraduate (6)	8.33±2.16	10.83±2.56	4.67±2.16	23.83±5.91
Physician (3)	6.33±1.53	9.33±1.53	3.00±1.73	18.67±3.21

Analysis of KAP scores across educational levels revealed important trends. Knowledge scores did not differ significantly between groups (H=5.44, p=0.245, η²=0.05, 95% CI: 0.00-0.11). Attitude scores, however, showed a significant difference (H=6.11, p=0.048, η²=0.07, 95% CI: 0.01-0.13). Post hoc Bonferroni-adjusted comparisons indicated that PGs (median: 5, IQR: 4-6) and physicians (median: 5, IQR: 4-6) had significantly higher attitude scores compared to 1st-year MBBS students (median: 4, IQR: 3-5; adjusted p=0.048). Practice scores demonstrated a non-significant trend (H=6.62, p=0.097, η²=0.06, 95% CI: 0.00-0.12), with physicians reporting the highest median practice score (4, IQR: 3-5), while 1st-year MBBS students had the lowest score (median: 2, IQR: 1-3). Total KAP scores followed a progressive pattern, with 1st-year students scoring lowest (18.08±5.15) and PGs scoring highest (23.83±5.91), reflecting incremental improvement with advancing education (Table 9). A pathway analysis diagram depicting KAP scores in relation to sex, age, and educational level is presented in Appendix 7.

**Table 9:** Comparison of KAP domains across educational levels among study participants (n=219). KAP: Knowledge, attitudes, and practices; H statistic: Kruskal-Wallis H statistic; p-value: Probability value; η²: eta-squared; adjusted p-value: Bonferroni-corrected probability value. Group differences were assessed using the Kruskal-Wallis H test for non-parametric data. Post hoc pairwise comparisons were performed using Dunn’s test with Bonferroni correction to adjust for multiple testing. Effect sizes were reported as eta-squared (η²), with 95% CIs used to quantify the magnitude of differences. A p-value of less than 0.05 was considered statistically significant.

Variable	Group	Median (IQR)	H statistic	p-value	Adjusted p-value (Bonferroni)	η² (effect size)	95% CI for η²
Knowledge	MBBS 1st and 2nd year	7 (6-9)	4.12	0.245	>0.05	0.04	-0.01-0.09
	MBBS 3rd and 4th year/intern	8 (7-10)					
	Postgraduates	9 (8-11)					
	Physicians	9 (8-12)					
Attitudes	MBBS 1st and 2nd year	3 (2-4)	6.11	0.048	0.048	0.07	0.02-0.12
	MBBS 3rd and 4th year/intern	4 (3-5)					
	Postgraduates	5 (4-6)					
	Physicians	5 (4-6)					
Practices	MBBS 1st and 2nd year	2 (1-3)	5.21	0.097	>0.05	0.05	0.00-0.10
	MBBS 3rd and 4th year/intern	3 (2-4)					
	Postgraduates	3 (2-4)					
	Physicians	4 (3-5)					

Table 10 shows the inter-domain correlation matrix among KAP scores. Knowledge was moderately correlated with attitude (ρ=0.42) and practice (ρ=0.36), while attitude demonstrated a moderate correlation with practice (ρ=0.48). All correlations were positive, indicating that higher knowledge scores were associated with more favorable attitudes and better preventive practices, and that positive attitudes were linked to stronger adoption of protective behaviors (Table 10).

**Table 10:** Inter-domain correlations between KAP scores among study participants (n=219). ρ: Spearman’s rank correlation coefficient; NA: Not applicable. Spearman’s rank correlation coefficient was used to assess the relationships between knowledge, attitude, and practice scores. ρ values were interpreted as follows: 0.10-0.29 = weak correlation, 0.30-0.49 = moderate correlation, and ≥0.50 = strong correlation.

Variables	Knowledge (ρ)	Attitude (ρ)	Practice (ρ)
Knowledge score	1	NA	NA
Attitude score	0.42	1	NA
Practice score	0.36	0.48	1

Table 11 presents Spearman’s rank correlation coefficients with 95% CIs to assess inter-domain KAP correlations. Knowledge was moderately correlated with attitude (ρ=0.40, 95% CI: 0.29-0.51, p<0.001) and weakly correlated with practice (ρ=0.28, 95% CI: 0.15-0.40, p<0.001). Attitude showed a moderate positive correlation with practice (ρ=0.33, 95% CI: 0.21-0.44, p<0.001) (Table 11). The correlation matrix between KAP scores and demographic variables is presented in Appendix 8.

**Table 11:** Inter-domain correlations between KAP scores among study participants (n=219). KAP: Knowledge, attitudes, and practices; ρ: Spearman’s rank correlation coefficient; p-value: Probability value. A p-value of less than 0.05 was considered statistically significant.

Domain pair	Spearman’s rho (ρ)	95% CI	p-value
Knowledge-attitude	0.4	0.29-0.51	<0.001
Attitude-practice	0.33	0.21-0.44	<0.001
Knowledge-practice	0.28	0.15-0.40	<0.001

## Discussion

**Figure 1:**
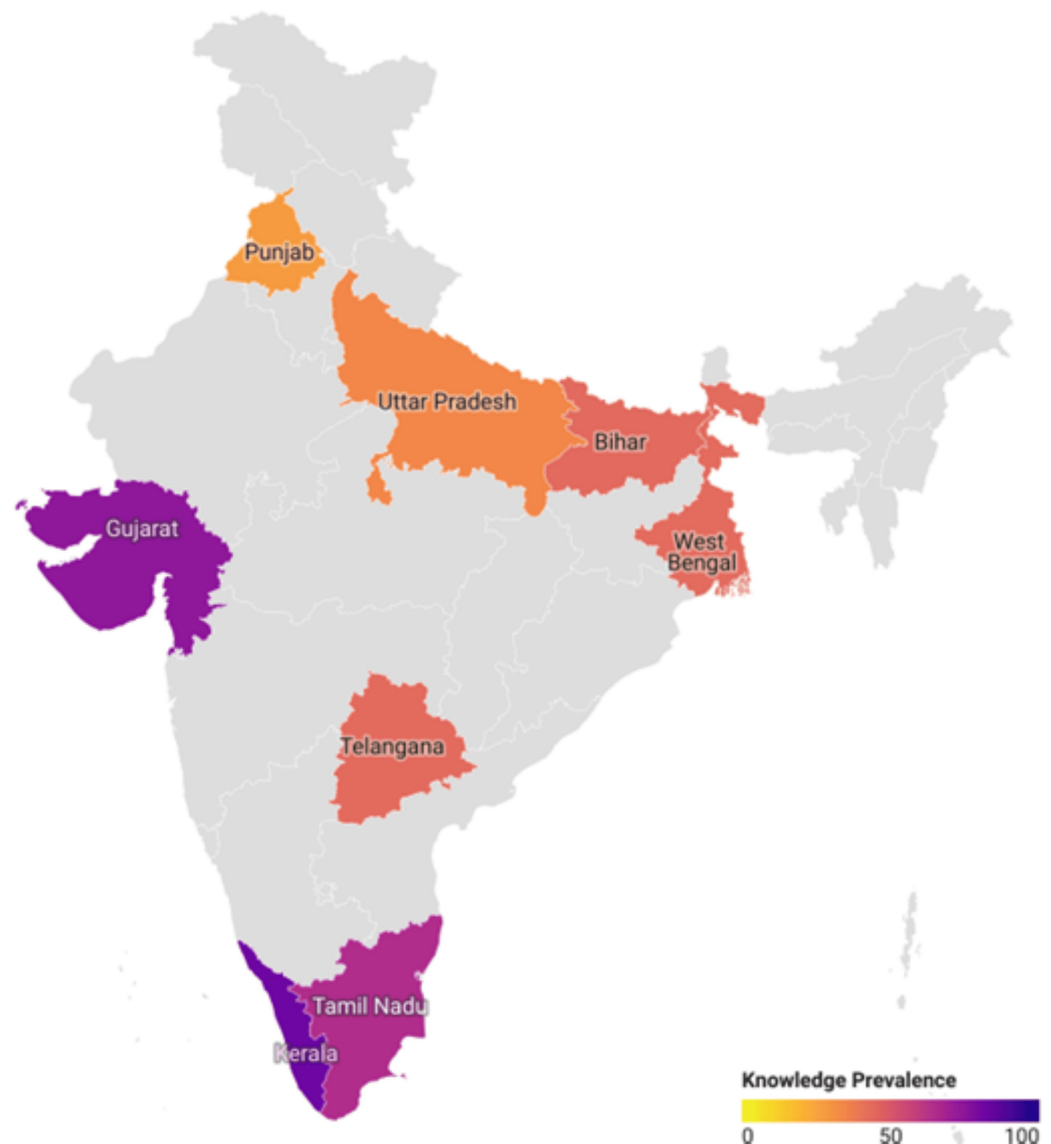
Proportion of mpox knowledge scores among healthcare professionals and students across India. The figure was created by the authors using Datawrapper (https://app.datawrapper.de/select/map). The figure was generated using data synthesized from references [14,15,18-21].

This study assessed KAP related to mpox among 219 medical students, interns, PG trainees, and physicians in Telangana. Participants demonstrated moderate knowledge (7.26±2.65 out of 15), moderate attitudes (4.19±1.51 out of 8), and critically low preventive practices (3.17±1.64 out of 8). The overall mean KAP score was 14.62±4.49 out of 31, underscoring limited preparedness among future healthcare professionals, particularly as most respondents were early-stage MBBS students with minimal clinical exposure. The moderate knowledge score highlights gaps in the UG curriculum, which does not adequately address contemporary information on mpox or other emerging zoonotic threats. These findings align with Bhadra A et al.’s study in Kolkata, where more than half of medical students had limited understanding of mpox [15], and with Alshahrani NZ et al.’s report of insufficient awareness among healthcare personnel in Saudi Arabia [16]. In contrast, Harapan H et al. documented strong knowledge among general practitioners in Indonesia [17]. The low awareness observed in India likely reflects the absence of structured institutional seminars and the historically low prevalence of mpox, leading to limited educational emphasis. Supporting evidence from Islam MA et al. and Gandhi RK et al. indicates that awareness is strongly shaped by educational background, reinforcing the importance of curriculum exposure to new diseases [8,14]. Regional disparities further illustrate this trend: states such as Gujarat, Kerala, and Tamil Nadu report higher knowledge scores, likely due to greater access to information and stronger institutional focus on emerging infectious diseases, whereas Telangana, Bihar, West Bengal, Punjab, and Uttar Pradesh show comparatively lower scores [18-21]. Collectively, these observations emphasize the need for regular curriculum updates and standardized training to strengthen preparedness among healthcare professionals (Figure 1).

Participants in this study demonstrated moderate to favorable attitudes toward mpox prevention (4.19±1.51), reflecting appreciation for timely medical intervention and preventive strategies. Similar findings were reported by Gandhi RK et al. in Gujarat, India [14], Zhou L et al. in China [22], and Islam MA et al. in Bangladesh [8], where constructive attitudes were likely influenced by the WHO’s declaration of mpox as a PHEIC. However, attitudes were not universally favorable; Amer FA et al. found that only 44.5% of medical professionals expressed positive opinions about mpox [23], suggesting that stigma and the lingering effects of the COVID-19 pandemic may contribute to variability.

Demographic analysis revealed no significant sex-based differences in knowledge, attitudes, or practices (all p>0.05). Age and educational level, however, were significantly associated with preparedness. Knowledge scores showed an overall difference across age groups (H=6.94, p=0.031), but this did not remain significant after post hoc adjustment (adjusted p=0.186). Attitudes (H=7.02, p=0.030, η²=0.08) and practices (H=7.64, p=0.022, η²=0.07) varied significantly. Participants aged ≥23 years (median attitude: 5, IQR: 4-6; median practice: 4, IQR: 3-5) had more positive attitudes (adjusted p=0.048) and higher practice scores (adjusted p=0.044) compared to the youngest group (17-19 years; median attitude: 3, IQR: 2-4; median practice: 2, IQR: 1-3). Pairwise comparisons showed that the 17-19-year group scored lower than the 20-22-year group in knowledge (p=0.011), attitude (p=0.037), and practice (p=0.029), and lower than the ≥23-year group in attitude (p=0.031) and practice (p=0.022). No significant differences were observed between the 20-22- and ≥23-year groups. These findings highlight the importance of academic progression in shaping preparedness, with the youngest cohort consistently performing worst.

Educational level comparisons reinforced this trend. First-year MBBS students had the lowest total KAP scores (18.08±5.15), while PG trainees achieved the highest scores (23.83±5.91). Knowledge scores increased progressively from 6.43±2.65 in 1st-year students to 8.33±2.16 in PGs, and practice scores peaked among PGs (4.67±2.16). Kruskal-Wallis analysis showed significant differences in attitudes (H=6.11, p=0.048, η²=0.07), with PGs and physicians scoring higher (median: 5, IQR: 4-6) than 1st-year students (median: 3, IQR: 2-4; adjusted p=0.048). Knowledge differences across educational levels were non-significant (H=4.12, p=0.245), while practice scores showed a non-significant trend (H=5.21, p=0.097, η²=0.05). Despite gains in knowledge and attitudes with academic advancement, practice scores remained the weakest domain, ranging from 2.78±1.20 in 4th-year students to 4.67±2.16 in PGs. This gap between theoretical understanding and preventive behavior has also been reported by Singh T and Sharma S in Bihar, India [18], and Bates BR and Grijalva MJ in Ohio, USA [24].

Correlation analysis revealed significant inter-domain associations. Knowledge correlated moderately with attitude (ρ=0.40, 95% CI: 0.29-0.51, p<0.001) and weakly with practice (ρ=0.28, 95% CI: 0.15-0.40, p<0.001). Attitude correlated moderately with practice (ρ=0.33, 95% CI: 0.21-0.44, p<0.001). Students relying on academic sources, such as lectures, textbooks, and faculty, achieved significantly higher KAP scores across all domains compared to those depending on social media or peers (p<0.05), underscoring the importance of formal educational channels. While increasing age was associated with more positive attitudes in the present study, Islam MA et al. and Abd ElHafeez S et al. reported stronger correlations between knowledge and age [8,25].

Despite moderate knowledge and positive attitudes, preventive practices remained inadequate. Contributing factors may include insufficient hands-on training, perceived low local risk of mpox, absence of institutional protocols, and limited vaccine access. Knowledge levels did not differ significantly across educational groups, indicating that awareness of emerging infectious diseases is not uniformly embedded in curricula [26]. Although older participants and advanced trainees demonstrated more positive attitudes, this did not consistently translate into improved practices, echoing earlier studies documenting a disconnect between knowledge and application [27]. Uniformly low practice scores across demographics reinforce concerns that medical students and young professionals lack opportunities for infection-control and outbreak-response training [28]. Structured interventions, such as simulation-based learning, outbreak preparedness modules, and mandatory reporting exercises, are therefore critical.

These findings align with global reports emphasizing the influence of ME and professional status on attitudes toward vaccination and public health measures [29]. The absence of sex-based differences suggests that educational exposure and clinical experience are stronger determinants of preparedness than demographic variables. Self-perceived knowledge did not consistently translate into preparedness, echoing evidence that confidence often overestimates competence [30]. Factors contributing to poor practice include limited awareness initiatives, lack of firsthand outbreak experience, and inadequate training opportunities. The youngest students (17-19 years) represent the most vulnerable cohort, underscoring the need to integrate preventive strategies early in medical training. Embedding applied infection-control measures and competency-based sessions into curricula is essential to bridge the gap between knowledge and practice. Collectively, these results highlight the urgent need to strengthen mpox-related education and training among medical students and professionals in India, ensuring preparedness through targeted interventions such as case-based discussions, outbreak drills, and structured public health exercises.

Study limitations

The primary limitations of this study stem from its cross-sectional design, which provides only a snapshot of mpox KAP at a single point in time and cannot capture changes in students’ understanding over time. Furthermore, the single-center setting limits the generalizability of the findings beyond the studied institution. A key constraint was the disproportionate sample size, with an overrepresentation of early-stage MBBS students and sparse inclusion of senior cohorts, including interns, PGs, and physicians, which limited the power to draw robust conclusions about the entire healthcare workforce. The reliance on self-administered questionnaires introduces the potential for social desirability and recall bias, which may have led to over-reporting of positive attitudes and knowledge. Finally, the use of purposive sampling, while appropriate for targeting the most relevant population, may contribute to selection bias and reduce the external validity of the outcomes.

## Conclusions

This study found that medical students in Telangana demonstrated moderate knowledge and favorable attitudes but critically low preventive practices regarding mpox. Key demographic factors, specifically increasing age and educational progression, were significantly associated with improvements in attitudes and practices, although a persistent gap between theoretical knowledge and practical application remains. The correlations among knowledge, attitudes, and practices underscore the potential for educational interventions to drive positive behavior. This inaugural study in Telangana highlights the urgent need for curricular reform to integrate comprehensive content on emerging zoonotic threats such as mpox. Future educational efforts must focus on moving beyond theoretical knowledge to competency-based, hands-on training to effectively translate positive attitudes into consistent and effective preventive practices, thereby strengthening the preparedness of future healthcare professionals against public health emergencies.

## Figures and Tables

**Figure 2:**
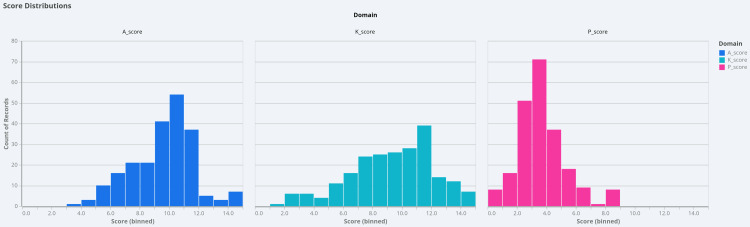
Bar diagram showing the distribution of KAP scores. KAP: Knowledge, attitudes, and practices.

**Figure 3:**
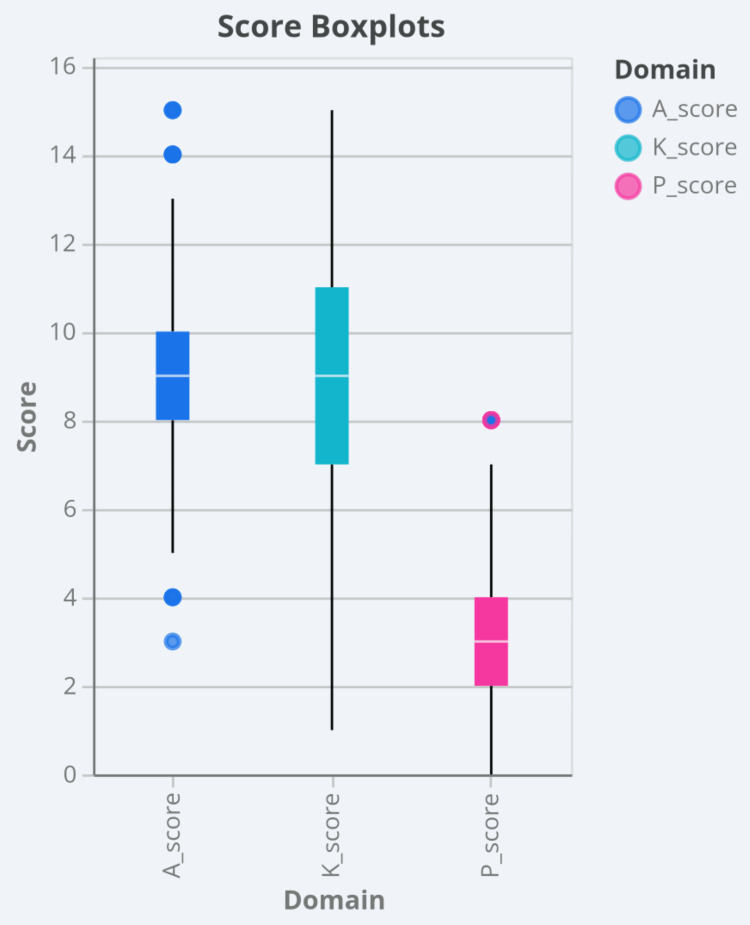
Distribution of KAP scores represented in a box plot. KAP: Knowledge, attitudes, and practices.

**Figure 4:**
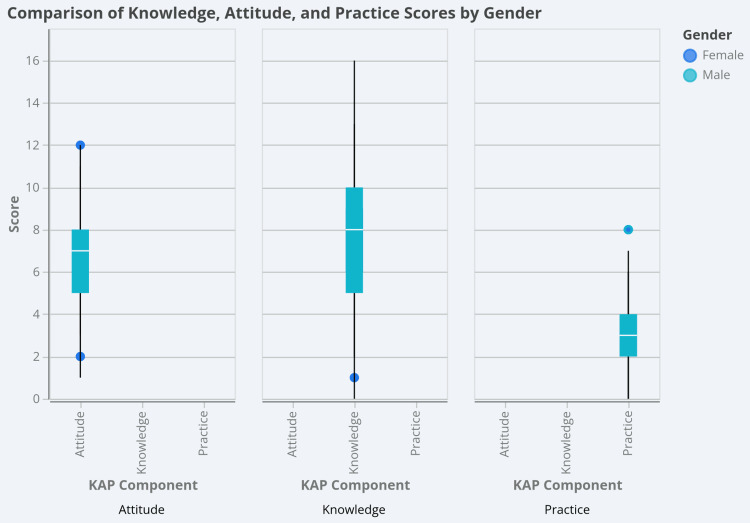
Graphical representation of sex-based KAP scores. KAP: Knowledge, attitudes, and practices.

**Figure 5:**
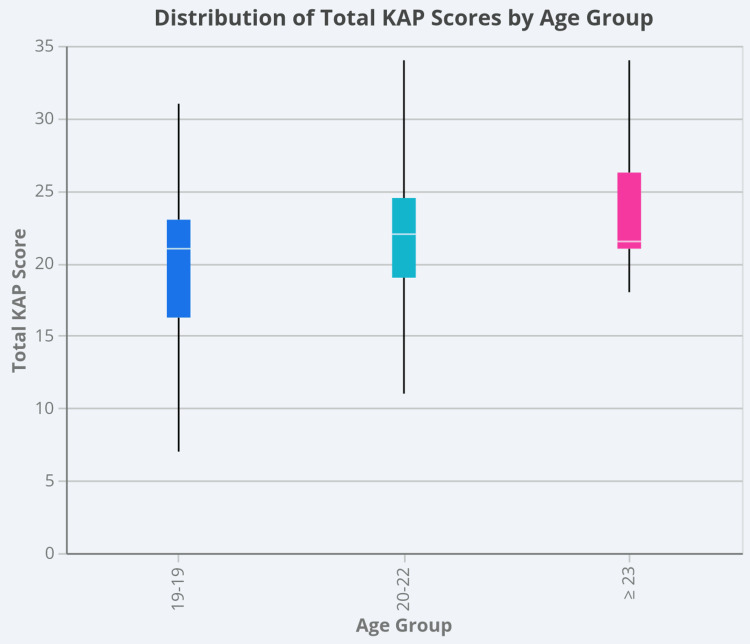
Graphical representation of age-based distribution of KAP scores. KAP: Knowledge, attitudes, and practices.

**Figure 6:**
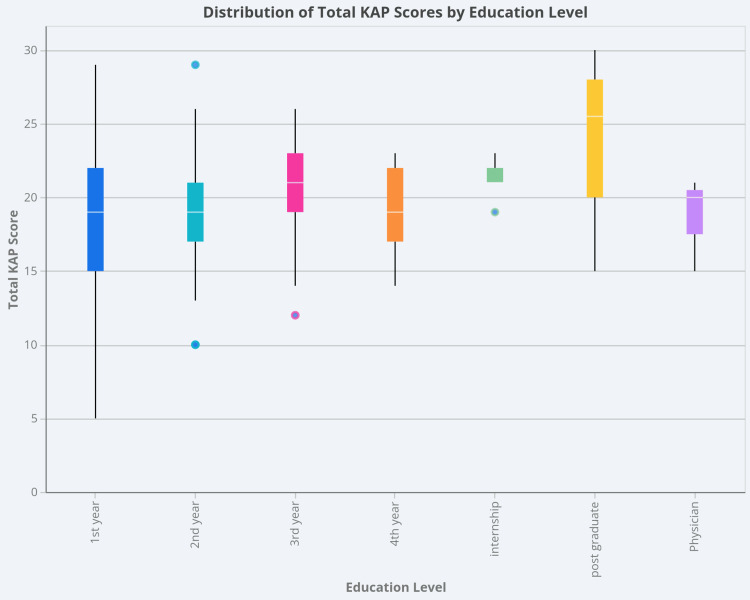
Graphical representation of KAP scores by educational level. KAP: Knowledge, attitudes, and practices.

**Figure 7:**
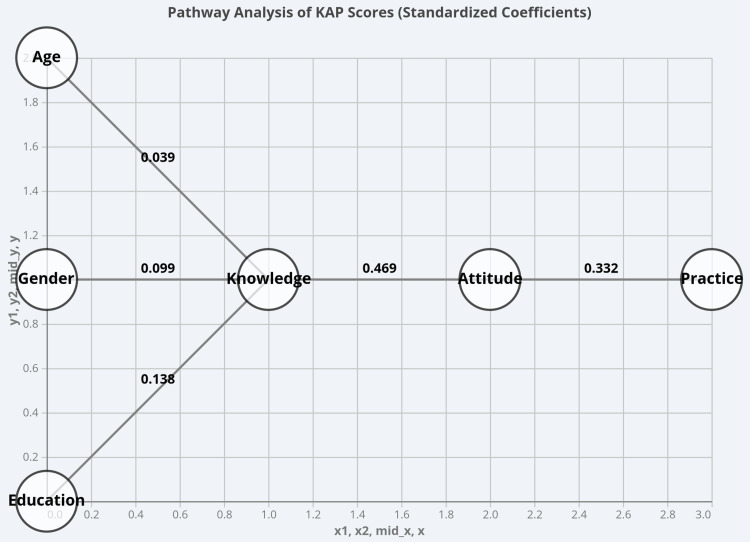
Pathway analysis diagram depicting KAP scores in relation to sex, age, and educational level. KAP: Knowledge, attitudes, and practices This image was created by the authors.

**Figure 8:**
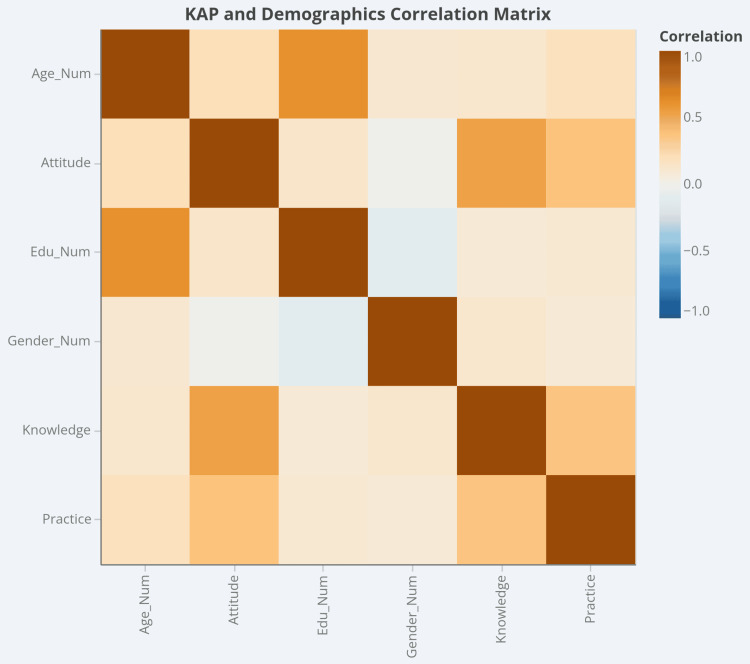
Correlation matrix between KAP scores and demographic variables. KAP: Knowledge, attitudes, and practices This image was created by the authors.

## Appendices

KAP on mpox questionnaire

Demographic details

1. What is your age?

☐ 17 years ☐ 18 years ☐ 19 years ☐ 20 years ☐ 21 years ☐ 22 years ☐ 23 years ☐ 24 years ☐ 25 years ☐ 26 years ☐ 27 years ☐ 28 years ☐ 29 years ☐ 30 years and above

2. Gender (check one)

☐ Male ☐ Female ☐ Other

3. What is your current level of medical education or professional status? (check one)

☐ 1st-year MBBS ☐ 2nd-year MBBS ☐ 3rd-year MBBS ☐ 4th-year MBBS ☐ Intern ☐ Postgraduate ☐ Physician

4. Which state or union territory are you from? (check one)

☐ Andhra Pradesh ☐ Telangana ☐ Karnataka ☐ Kerala ☐ Tamil Nadu ☐ Bihar ☐ Delhi ☐ Haryana ☐ Himachal Pradesh ☐ Jammu and Kashmir ☐ Jharkhand ☐ Madhya Pradesh ☐ Punjab ☐ Rajasthan ☐ Uttar Pradesh ☐ Uttarakhand ☐ Assam ☐ Odisha ☐ West Bengal ☐ Chhattisgarh ☐ Goa ☐ Gujarat ☐ Maharashtra ☐ Arunachal Pradesh ☐ Manipur ☐ Meghalaya ☐ Mizoram ☐ Nagaland ☐ Sikkim ☐ Tripura ☐ Andaman and Nicobar Islands ☐ Chandigarh ☐ Dadra and Nagar Haveli and Daman and Diu ☐ Ladakh ☐ Lakshadweep ☐ Puducherry

Knowledge

5. Have you heard about mpox virus infection?

☐ Yes ☐ No

6. What is the mode of transmission of mpox? (check all that apply)

☐ Skin-to-skin contact, such as touching or sexual contact ☐ Mouth-to-mouth or mouth-to-skin contact, such as kissing ☐ Face-to-face contact with someone who has mpox, such as talking or breathing close to one another ☐ Mother-to-baby transmission ☐ Animal-to-human transmission

7.  What are the common symptoms of mpox? (check all that apply)

☐ Rash over the palms, soles, face, mouth, groin, and genital areas ☐ Fever ☐ Sore throat ☐ Headache ☐ Muscle pain ☐ Back pain ☐ Low energy ☐ Swollen lymph nodes

8. Is mpox a variant of smallpox?

☐ Yes ☐ No ☐ I don’t know

9. Is mpox a new variant of COVID-19? ☐ Yes ☐ No ☐ I don’t know

10. Is mpox a zoonotic disease?

☐ Yes ☐ No ☐ I don’t know

11.  Do you think mpox is a highly contagious disease? ☐ Yes ☐ No

12. Are healthcare workers at higher risk of contracting mpox due to occupational exposure? ☐ Yes ☐ No

13. Can mpox be fatal? ☐ Yes ☐ No

14. Is mpox a public health emergency? ☐ Yes ☐ No

15. Has your college, university, or workplace educated you about mpox? ☐ Yes ☐ No

16. Is mpox associated with typical skin lesions? ☐ Yes ☐ No ☐ I don’t know

17. Can mpox survive for several days on contaminated surfaces? ☐ Yes ☐ No ☐ I don’t know

18. Is there a specific treatment approved for mpox infection? ☐ Yes ☐ No ☐ I don’t know

19. Is there currently an approved vaccine for the prevention of mpox? ☐ Yes ☐ No ☐ I don’t know

20. Effective prevention strategies for mpox include: (check all that apply) ☐ Isolation of infected individuals ☐ Vaccination against mpox ☐ Conducting public health awareness camps ☐ Strengthening surveillance and contact tracing

Attitudes

21. How confident are you in identifying mpox symptoms? (select one) ☐ Not confident ☐ A little confident ☐ Very confident

22. Do you believe mpox is a public health threat like the COVID-19 pandemic? ☐ Yes ☐ No ☐ I don’t know

23. If the mpox vaccine is offered, would you be willing to receive it for your health and safety? ☐ Yes ☐ No ☐ I don’t know

24. Do you believe you have sufficient knowledge about mpox? ☐ Yes ☐ No ☐ I don’t know

25. Are you worried about the spread of mpox in India and your local area? ☐ Yes ☐ No

26. Do you think the Indian government is adequately prepared to handle mpox outbreaks? ☐ Yes ☐ No ☐ I don’t know

27. Do you believe vaccination against mpox is essential, particularly for healthcare professionals? ☐ Yes ☐ No ☐ I don’t know

28. Training and education on mpox are essential for all medical students and healthcare professionals. ☐ Strongly agree ☐ Agree ☐ Neutral ☐ Disagree ☐ Strongly disagree

Practices

29. Have you attended any training or workshop on mpox? ☐ Yes ☐ No

30. Do you routinely screen patients with febrile illness for recent travel history or exposure to potential zoonotic diseases? ☐ Yes ☐ No ☐ I don’t know

31. Are you familiar with the local protocol for notifying health authorities about a suspected mpox case? ☐ Yes ☐ No

32. Do you always follow proper hand hygiene protocols at your workplace? ☐ Yes ☐ No

33. If a confirmed mpox case were identified in your community, would you volunteer to assist in patient management or contact tracing? ☐ Yes ☐ No ☐ Not sure

34. Do you encourage others to follow public health guidelines regarding mpox prevention? ☐ Yes ☐ No

35. Have you participated in any community awareness programs on mpox? ☐ Yes ☐ No

36. Do you think your current healthcare facility is adequately prepared to handle an mpox outbreak? ☐ Yes ☐ No ☐ Not sure

Appendix 1

To ensure transparent and standardized reporting of observational research, the Strengthening the Reporting of Observational Studies in Epidemiology (STROBE) checklist was applied to this study. The checklist provides a structured framework for evaluating the completeness of reporting across key domains, including the title, abstract, introduction, methods, results, discussion, and other information. Incorporating the STROBE checklist highlights how the present cross-sectional study on knowledge, attitudes, and practices concerning mpox among medical students in Telangana aligns with established reporting guidelines, while also identifying areas for improvement.

**Table 12:** STROBE checklist for cross-sectional studies applied to the mpox KAP study. STROBE: Strengthening the Reporting of Observational Studies in Epidemiology; KAP: Knowledge, attitudes, and practices; IEC: Institutional Ethics Committee; PHEIC: Public Health Emergency of International Concern.

Section	Item	Recommendation	How addressed in the paper
Title and abstract	1	Indicate the study design in the title and/or abstract	The title specifies “cross-sectional analysis,” and the abstract clearly states the study design.
Introduction	2	Background/rationale	The paper provides context on mpox as a PHEIC and explains the importance of medical students in outbreak preparedness.
	3	Objectives	The paper states the primary and secondary hypotheses and links the study objectives to STROBE reporting.
Methods	4	Study design	A cross-sectional descriptive study was conducted at PIMS, Telangana.
	5	Setting	The location, timeframe (December 2024-May 2025), and institutional context are described.
	6	Participants	Inclusion/exclusion criteria, purposive sampling, consent, and IEC approval are described.
	7	Variables	KAP domains, scoring thresholds, and demographic variables are defined.
	8	Data sources/measurement	A validated 32-item questionnaire was used; pilot testing was conducted, and Cronbach’s α was reported.
	9	Bias	Possible social desirability and recall bias are mentioned in the limitations, but mitigation strategies during data collection are not described.
	10	Study size	The sample size calculation formula is provided, with a final sample size of n=219.
	11	Quantitative variables	Scoring is explained, including Likert scales, thresholds, and classification of KAP levels.
	12	Statistical methods	Non-parametric tests, including the Kruskal-Wallis test, Dunn’s test, Mann-Whitney U test, and Spearman’s correlation, were used; effect sizes and CIs were reported.
Results	13	Participants	Participant flow is described: 220 estimated, 219 completed, and a 99.5% response rate.
	14	Descriptive data	Sociodemographic data, including age, sex, educational level, and state of residence, are presented in Table 1.
	15	Outcome data	KAP scores are summarized in Tables 2-11 and relevant figures.
	16	Main results	Age- and education-based differences and correlations are reported with p-values and η² values.
	17	Other analyses	Subgroup comparisons by sex, age, and educational level, along with correlation matrices, are presented.
Discussion	18	Key results	The paper summarizes moderate knowledge, favorable attitudes, and low preventive practices.
	19	Limitations	The paper notes the cross-sectional design, single-center setting, sampling bias, and self-report bias.
	20	Interpretation	Findings are compared with other Indian and global studies, and curricular gaps are emphasized.
	21	Generalizability	Limited external validity due to the single-institution setting is acknowledged.
Other information	22	Funding	The funding statement is provided.
Additional ethical information	Not a STROBE item	Ethical approval and consent	IEC approval is cited (IEC/PIMS/UG/2024/12), and informed consent was obtained.

Appendix 2 

Appendix 3 

Appendix 4 

Appendix 5 

Appendix 6

Appendix 7 

Appendix 8
